# The Influence of Silica Fly Ash and Wood Bottom Ash on Cement Hydration and Durability of Concrete

**DOI:** 10.3390/ma17164031

**Published:** 2024-08-13

**Authors:** Jurgita Malaiškienė, Marija Vaičienė

**Affiliations:** 1 Laboratory of Composite Materials, Institute of Building Materials, Faculty of Civil Engineering, Vilnius Gediminas Technical University, Linkmenu st. 28, LT-08217 Vilnius, Lithuania; 2 Civil Engineering Faculty, Vilnius College of Technologies and Design, Antakalnio st. 54, LT-10223 Vilnius, Lithuania; m.vaiciene@vtdko.lt

**Keywords:** silica fly ash, wood waste bottom ash, cement hydration, properties of concrete, durability of concrete

## Abstract

This research addresses a notable gap in understanding the synergistic effects of high carbon wood bottom ash (BA) and silica fly ash (FA) on cement hydration and concrete durability by using them as a supplementary material to reduce the amount of cement in concrete and CO_2_ emissions during cement production. This study analyses the synergistic effect of FA and BA on cement hydration through X-ray diffraction (XRD), thermal analysis (TG, DTG), scanning electron microscopy (SEM), density, ultrasonic pulse velocity (UPV), compressive strength, and temperature monitoring tests. In addition, it evaluates concrete properties, including compressive strength, UPV, density, water absorption kinetics, porosity parameters, predicted resistance to freezing and thawing cycles, and results of freeze–thawing resistance. The concrete raw materials were supplemented with varying percentages of BA and FA, replacing both cement and fine aggregate at levels of 0%, 2.5%, 5%, 10% and 15%. The results indicate that a 15% substitution of BA and FA delays cement hydration by approximately 5 h and results in only a 6% reduction in compressive strength, with the hardened cement paste showing a strength similar to a 15% replacement with FA. Concrete mixtures with 2.5% BA and 2.5% FA maintained the same maximum hydration temperature and duration as the reference mix. Furthermore, the combined use of both ashes provided adequate resistance to freeze–thaw cycles, with only a 4.7% reduction in compressive strength after 150 cycles. Other properties, such as density, UPV and water absorption, exhibited minimal changes with partial cement replacement by both ashes. This study highlights the potential benefits of using BA and FA together, offering a sustainable alternative that maintains concrete performance while using waste materials.

## 1. Introduction

Carbon emissions contribute significantly to global warming, prompting nations to address this issue urgently. The rising demand for cement exacerbates the problem, but the use of supplementary cementitious materials (SCMs), which act as fillers and leverage their pozzolanic activity, can be adopted as a solution to reduce cement-related carbon emissions [1,2,3]. Aligned with the objectives of the circular economy, the conversion of waste streams into valuable resources is a central goal. Ash, a major industrial by-product, should be prioritised for applications beyond current landfilling practices. The use of ash is of paramount importance for advancing the circular economy and reducing landfill disposal [4]. To address the environmental impacts of cement manufacturing, such as the depletion of natural resources, it is crucial to strengthen the use of SCMs to ensure sustainable concrete production. There is also an increasing global demand for carbon-efficient solutions that reduce CO_2_ emissions and utilise waste, aligning with the Paris Agreement’s goal to limit global temperature rise to 1.5 °C. Fly ash, an industrial by-product, has been used to replace cement and produce more durable and cost-effective construction materials. However, the availability of fly ash is decreasing due to the closure of coal-based thermal power plants worldwide, though some of them are still operating and the amount in the landfills of such fly ash is still high. In the future, biomass ash could be a sustainable alternative to fly ash [5,6,7,8]. Currently, only Lithuania operates more than 160 biofuel boilers, consuming approximately 1.2 million cubic metres of wood annually. It is estimated that this results in the generation of 25,000 to 30,000 tons of ash per year. The recycling process for this ash is highly complex. Therefore, it is imperative to develop environmentally sustainable and economically viable solutions for recycling these by-products [9].

The use of coal fly ash and wood bottom ash as supplementary cementitious materials for partial replacement of cement in concrete offers several economic, technical, and environmental benefits, including the conservation of natural resources and the reduction in greenhouse gas emissions in the cement industry [10]. Physical, chemical, and morphological properties of various types of fly ash (FA) and bottom ash (BA) can vary significantly due to a variety of factors, including the composition of raw coal and/or biomass, the degree of pulverisation, combustion temperatures, methods, and storage conditions [11,12,13,14]. In terms of the reported calcination temperatures [15], carbonates and bicarbonates, particularly calcite (CaCO_3_), which are abundant in wood ash, can be obtained at low temperatures of about 500 °C. Calcite usually has an accelerating effect on cement hydration [16,17].

Fly ash, a by-product of coal combustion in power plants, can be used effectively to reduce the carbon footprint of the concrete industry by partially substituting Portland cement, which is known for its high energy consumption and associated CO_2_ emissions [15,18,19]. Moreover, the use of FA helps to conserve natural resources by lowering the demand for raw materials and reducing landfill waste [20]. Composed primarily of silica, alumina and calcium, FA contributes to the pozzolanic reaction when mixed with water and reacts with calcium hydroxide (CH) released during cement hydration. This reaction forms additional calcium silicate hydrate (CSH), which enhances the strength and durability of concrete [21]. The addition of fly ash not only improves the mechanical properties of concrete but also enhances long-term performance by reducing permeability and increasing resistance to chemical attacks [22]. The authors found [23] that FA improves the durability of concrete by reducing permeability and increasing resistance to attacks with sulphate and chloride due to the pozzolanic reaction, which reduces the presence of calcium hydroxide and makes the modified concrete less prone to chemical deterioration. The compressive strength of fly ash concrete has been the focus of attention in many studies. Research by Hashmi et al. [24] demonstrated that concrete with 25% replacement of cement with FA at 150 days exhibited compressive strength values comparable to reference concrete due to ongoing pozzolanic reactions. Similarly, Liu et al. [21] found that cement replacement with FA at 15–30% resulted in compressive strengths ranging from 30 MPa to 50 MPa, depending on the curing conditions and the design of the mix. It should be noted [25] that cement replacement with 20–30% FA resulted in the maximum compressive strength of ultra-high-performance concrete developed at a later age of 90 and 180 days, but the early compressive strength of the concrete with FA was less than that of the reference specimen. It was found [26,27,28], that the effect of pozzolanic activity of FA develops slowly and thus, during the first few days, the compressive strength of the cement-based material reduces in comparison with the reference. The reduction in compressive strength is more expressed in the specimens with a higher ash content. Furthermore, with a constant FA content, a reduction in the water-to-binder ratio from 0.18 to 0.12 led to an increase in the compressive strength from 9% to 57%, with higher FA contents producing more pronounced positive effects. Remarkably, the incorporation of 70% FA significantly increased the compressive strength of concrete specimens by 50–57% compared to the specimens containing 20–50% FA, which showed an improvement of only 9% to 23%. FA with fine particles and spherical shape could improve concrete workability, reduce water demand, and mitigate issues related to thermal cracking [29]. However, the properties of FA can vary significantly depending on factors such as the source of the coal, the combustion conditions, and the collection methods, affecting its performance in concrete [30]. Understanding these variations is essential for optimising the use of FA in concrete mixtures and ensuring consistent quality and performance.

Similar to FA, wood bottom ash concrete often exhibits reduced early-age strength. This delayed pozzolanic reaction is crucial for long-term strength gain and durability [31]. The larger particle size and variability in composition can contribute to this reduction. Over time, the pozzolanic activity of wood ash, activated by the hydration products of Portland cement, can improve strength. Research has shown that concrete with BA, as with FA, can achieve strength gains comparable to or exceeding reference concrete by 90 days [32]. BA is also rich in oxides such as silica, alumina, and calcium, which are essential for the hydration and strength development [32]. Its incorporation into concrete has been found to influence various properties, including workability, setting time, compressive strength, and durability. However, the performance of BA in concrete is highly dependent on its chemical composition and physical characteristics, which can vary widely due to different factors, such as biomass type, combustion conditions, and post-combustion processing [33]. Previous research has shown that wood ash can enhance the pozzolanic activity in concrete, particularly during the later stages of curing, due to its ability to react with CH to form additional calcium aluminate hydrates (CAH) and calcium silicate hydrate (CSH) gels [34,35,36]. This gel formation filled the voids within the matrix, thereby refining the pore structure and decreasing the porosity. Additionally [37], the improved quality of the interaction zone ensured a stronger bond between the cement paste and the aggregates, leading to better stress distribution and increased resistance to mechanical stresses. These combined effects of microstructural improvements ultimately improved both the mechanical strength and the longevity of the concrete. However, the variability in composition can impact its effectiveness. Proper characterisation and mix design are essential to achieve consistent results [33]. The incorporation of BA into cement composites causes an increase in water demand [38], primarily due to several key factors. These include the irregular particle shape and fineness of BA, the presence of free calcium oxide (CaO), and alkali content, as well as the loss of ignition (LOI) values. The irregular morphology and fine particles of BA increase the specific surface area, thereby necessitating a higher water content for adequate workability. Furthermore, the high levels of free CaO and alkalis in BA can lead to greater water absorption and chemical interactions within the mix, which further contributes to the increased demand for water [38]. The use of fly ash as a fine and/or coarse aggregate in concretes has received considerable attention in various countries with the aim of broadening the range of potential applications of BA in the construction sector [39,40,41,42]. According to researchers, BA is a new material with high potential. The use of BA in the concrete industry would not only reduce its disposal in landfills but would also make concrete production more sustainable. There are recent reports on the use of BA for various applications, such as masonry products, low- and medium-strength concrete, road paving systems, cementitious mixes and as an alternative aggregate in mortars [43,44,45,46]. The researchers [47] tested concrete where conventional Portland cement was replaced by weight with 0%, 20%, 50%, 75%, and 100% bottom ash and 20% by weight with coal fly ash. No significant effect on the compressive strength of all specimens was observed at 28 days. Other authors reported that FA and BA had a similar impact on cement hydration, depending on the particle size, but the effect of both types of ash on the workability of the fresh cement paste and other properties of hardened cement paste was different. Future research should focus on additives to mitigate the negative effects of FA and BA, enhancing the strength and durability by reducing the use of cement and natural aggregates.

The studies by Zhang et al. [48] and Wu et al. [49] highlight the significant benefits achieved through the synergistic effects of combining multiple industrial by-products (converter steelmaking slag, blast furnace slag, Bayer red mud, fly ash and desulfurization gypsum). This approach not only improves material performance but also supports environmental sustainability by promoting effective waste recycling.

There is a significant research gap to understand the synergistic effects of fly ash (FA) and wood bottom ash (BA), especially those with a high carbon and carbonates content, on the hydration, physical-mechanical properties, and freeze–thaw resistance of cement-based materials. This study addresses this gap by investigating the impact of substituting up to 30% of cement with a mixture of FA and BA, with the aim of providing new insights into their synergistic effects on cement composites. Additionally, in the concrete a part of the cement was replaced by FA and a part of the sand by BA.

## 2. Materials and Methods

Cement specimens were prepared for thermogravimetric (TG) and X-ray analysis (XRD) from cement CEM II/A-LL 42.5N, silica fly ash (FA) and wood waste bottom ash (BA). The chemical composition of these materials determined by the XRF method with the Rigaku ZSX IV spectrometer (Rigaku, Tokyo, Japan) is presented in Table 1.

**Figure 1:**
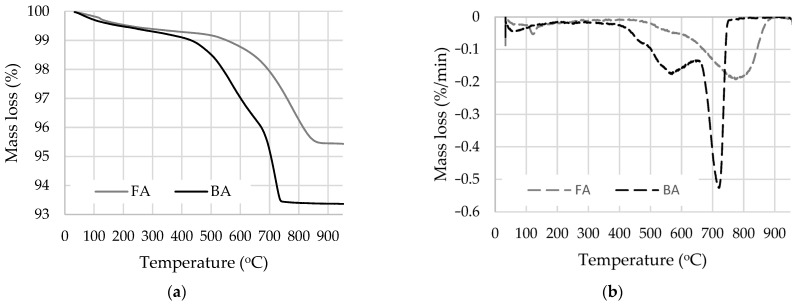
TG (**a**) and DTG (**b**) analysis of the FA and BA.

FA consists of SiO_2_ and Al_2_O_3_ (79.9%) and BA consists of SiO_2_, CaO, and CO_2_ (87.2%, the most in carbonates composition, because LOI in both ashes is about 4%). The fly ash is classified as F because it has a total oxide content of SiO_2_ + Al_2_O_3_ + Fe_2_O_3_ > 70%. Bottom ash contains a high amount of carbon (56.1% of CO_2_). The chemical composition of these types of ash differs greatly, and the influence on cement hydration could be different. Thermal analysis (TG, DTG) of BA and FA was determined on the thermal analyser Perkin Elmer TGA 4000 (Perkin Elmer, Waltham, MA, USA). The specimens having the weight of about 50 mg were placed into a platinum crucible and heated in nitrogen up to 980 °C at the heating rate 10 °C/min. The nitrogen was used to avoid additional combustion due to oxidation. The TG (Figure 1a) and DTG (Figure 1b) analysis of the ashes showed significant differences: BA has a much higher mass loss and two peaks in temperature ranges 420–650 °C and 650–750 °C which in these temperature ranges serve to decompose Ca(OH)_2_, and burn pure coal and various crystallinity carbonates, respectively. These results clarify the carbon compound in BA, which in chemical composition was shown to be CO_2_. FA has the highest mass loss in the temperature range 600–900 °C due to the decomposition of low- and high-crystallinity carbonates and this result showed in FA also, but in lower quantities and some of them have a higher crystallinity degree.

**Figure 2:**
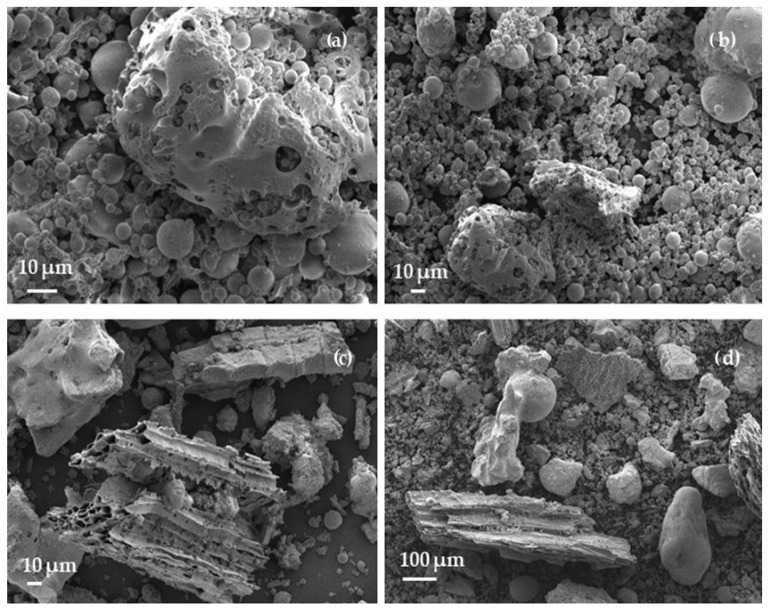
SEM images of FA and BA: (**a**,**b**)—FA and (**c**,**d**)—BA.

Microstructural analysis of BA and FA powders and the fractured surface of hardened cement paste samples was performed using a JSM-7600F (JEOL, Tokyo, Japan) scanning electron microscope. The voltage of 10 kV and the secondary electron mode were used for image formation. Before the test, the surface to be investigated was covered with a layer of electrically conducting material using a QUORUM Q150R ES device (Quorum Technologies Ltd., Lewes, UK). According to Figure 2, FA (Figure 2a,b) consists primarily of round particles 5–20 µm, but there are some porous agglomerates, which can have a higher water demand in the cementitious matrix. BA consists of three different types of particles (Figure 2c,d): larger elongated porous carbon particles up to 400 µm long, smaller particles up to 100 µm diameter, and various irregularly shaped particles 1–200 µm.

The particle size distribution of the materials was investigated using laser diffraction (Cilas 1090, Odelzhausen, Germany). The cement had an average particle size of 15.9 µm, FA of 36.6 µm and BA of 102 µm. The FA obtained from the coal-based thermal power plant in Ostrołęka (Ostroleka C Power Plant, Ostrołęka, Poland) is made of vitrified spherical fine grains. BA obtained by burning wood residues at 800 °C for 30 min was used for the tests.

The replacement of cement for the TG tests was as follows: 5% FA (FA5), 15% FA (FA15) and 5% FA + 5% BA (FBA5), 15% FA + 15% BA (FBA15). Thermal analysis (TG, DTG) was carried out at 28 days on the Perkin Elmer TGA 4000 (Perkin Elmer, Waltham, MA, USA). The samples of 50–60 mg weight were placed into a platinum crucible and heated in nitrogen up to 980 °C at a heating rate 10 °C/min. The content of portlandite (CH) and carbonates (CaCO_3_) in the tested specimens were calculated according to the method presented in [50] and then recalculated according to the amount of cement. XRD analysis was performed for 3 compositions (reference, FA15, FBA15) using a DRON-7 diffractometer (Bourevestnik, Saint Petersburg, Russia) with Cu-Kα (λ = 0.1541837 nm). The following test parameters were used: 30 kV voltage; 12 mA current; 2θ diffraction angle range from 4° to 60° with increment of 0.02° measured each 0.5 s.

The rheological properties of the cement paste were tested using a cylinder with a diameter of 3 cm and a height of 5 cm, according to EN 12706. The slump flow measurement was started 5 min after mixing. Specimens of 40 × 40 × 40 mm^3^ size were prepared and cured for 24 h at a temperature of 20 ± 1 °C and 95% relative humidity. The compositions are presented in Table 2. The demoulded samples were kept in water of 20 ± 1 °C for 7 or 28 days until the test. At 7 and 28 days, the density of the hardened cement paste samples was tested by measuring the dimensions with a precision of 0.01 mm and weighing with a precision of 0.01 g. Subsequently, the ultrasonic pulse velocity (UPV) was measured with a Pundit 7 device with two 54 kHz transducers according to the method described in [51], and the samples were compressed using the Tinius Olsen H200 KU press (Tinius Olsen, Orlando, FL, USA), according to the requirements of LST EN 196-1. Three samples of each composition were used for these tests.

The exothermic temperature was measured for the samples with a fine aggregate (sand 0/4 fr.) by recording the temperature rise under semi-adiabatic conditions. The 1300 g of prepared mixtures (Table 3) were poured into 100 × 100 × 100 mm^3^ moulds and a thermocouple in a glass pipe was inserted approximately into the middle of each sample to record the temperature. Thereafter, the mould was immediately placed in the container insulated with polystyrene foam. The experiments were carried out at (20 ± 1) °C and the temperature was recorded uninterruptedly until the temperature considerably decreased, i.e., at max. 48 h.

The concrete compositions are presented in Table 4. Characteristics of sand used for the tests: fraction 0–2 mm, bulk density 1.6 g/cm^3^. Crushed granite of fractions 4–16 mm was used as a coarse aggregate. A superplasticiser (SP) based on polycarboxylate ether polymers (Master Glenium Ace 560) was added to all mixtures. Characteristics of SP: density 1.06 g/cm^3^, pH 5.6 (20% solution at 20 °C). The mixtures for all specimens were made with tap water. Characteristics of polypropylene (PP) fibre Durus EasyFinish: density 0.922 kg/dm^3^, equivalent fibre diameter 0.7 mm, length 40 mm, ultimate tensile strength 500 MPa, complying with EN 14889-2 requirements. 100 × 100 × 100 mm^3^ concrete cubes were moulded from the mixtures under normal conditions. They were kept in steel moulds for 1 day and then soaked in water at 20 ± 2 °C for 27 days. Concrete samples were made and cured for strength tests according to EN 12390-2. The compressive strength of the hardened specimens was tested according to EN 12390-3 with a hydraulic press ALPHA 3-3000S test machine (FORM + TEST Seidner + Co. GmbH, Riedlingen, Germany) and density was measured according to EN 12390-7.

To determine the water absorption kinetics [52], the samples were dried to constant mass, weighed, immersed in water and weighed in air after 10 min, 30 min, 60 min, 24 h, and 48 h. The open porosity parameters of the concrete were determined by measuring water absorption. The resistance to freeze–thaw of the concrete was predicted based on the known porosity parameters of concrete and the methodology described in the articles [53,54].

The resistance to freeze–thaw cycles of concrete specimens (100 × 100 × 100 mm^3^) was determined after 28 days of curing according to the requirements of LST 1428-17. The remaining concrete samples were placed in the Rumed 3301 climate chamber (Rubarth Apparate GmbH, Laatzen, Germany). The rapid method was used by freezing the water-saturated concrete specimens in air and thawing them in water. For rapid freezing and thawing, the samples were soaked in 3% NaCl solution prior to testing [55].

## 3. Results

### 3.1. Influence of Fly Ash and Bottom Ash on the Properties of Hardened Cement Paste

**Figure 3:**
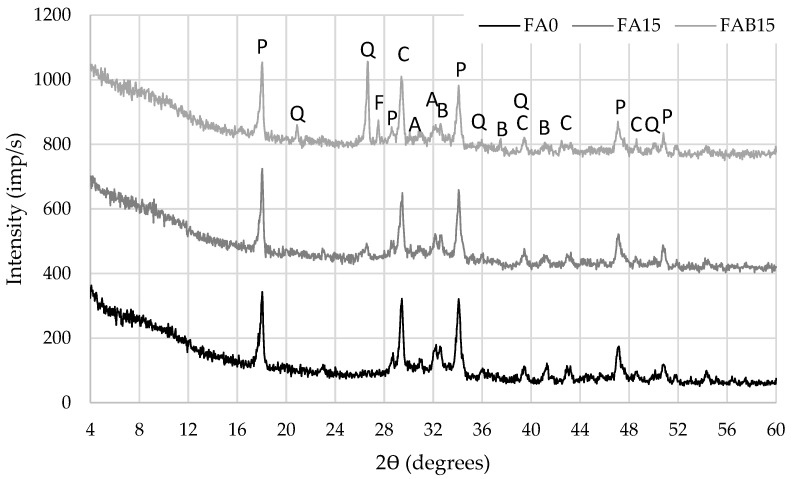
X-ray diffraction patterns of hardened cement paste after 28 days.

XRD tests showed (Figure 3) that samples from all compositions contained minerals common to all cement-based materials: portlandite (P), calcite (C), alite (A), belite (B), feldspar (F) and quartz (Q). Quartz was identified in all samples modified with FA and BA, but the highest intensity was observed in FBA samples containing 15% FA and 15% BA due to a high content of SiO_2_ in these ashes, 54.6% and 20.2%, respectively (Table 1). Although the CaO content in the cement mixes was different, the intensity of the portlandite and calcite peaks in all samples was similar, presumably due to the different levels of cement mineral hydration.

**Figure 4:**
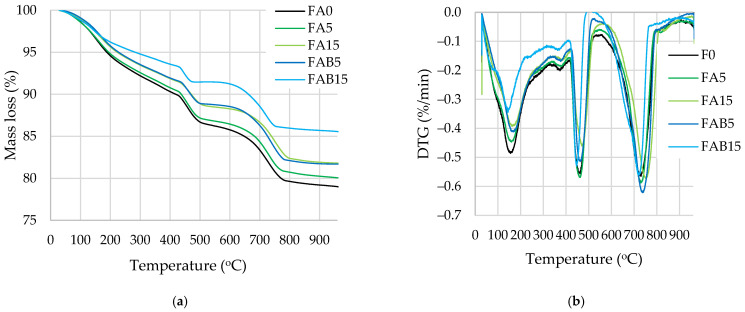
The TG (**a**) and DTG (**b**) curves of hardened cement paste after 28 days.

**Figure 5:**
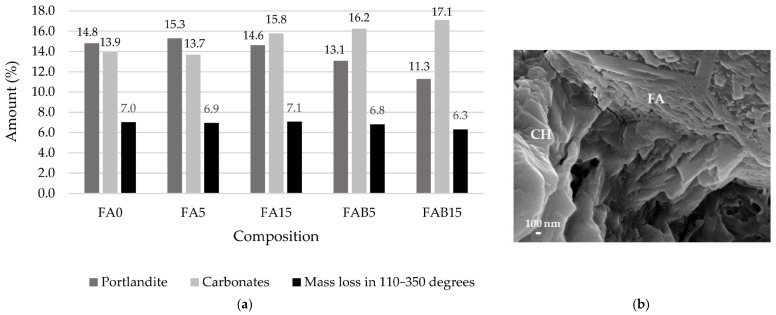
Amount of portlandite, calcite and mass loss in the temperature range of 110–350 °C (**a**) and SEM image of FA5 (**b**).

Thermal analysis was performed to identify the amount of hydration products, especially portlandite and calcite, after 7 days of curing. The mass loss (Figure 4 and Figure 5) in the temperature range from 30 °C to 110 °C is related to water evaporation; in the range from 110 °C to 350 °C the mass loss is caused by the dehydration of cement hydration products, including CSH, CASH, and ettringite; in the range between 420 °C and 530 °C the mass loss is related to the decomposition of portlandite and pure coal combustion (according to LOI (Table 1) about 4% in both ashes); and in the range between 610 °C and 800 °C the mass loss is caused by the decomposition of calcium carbonate of varying crystallinity [56,57]. Since the loss of water in several cement hydration products is recorded in the same temperature range (110–350 °C) and is mainly dependent on the drying conditions prior to TG analysis [58], the accurate quantification can only be achieved for products with well-defined nonoverlapping peaks, i.e., for portlandite and calcium carbonate [50].

**Figure 6:**
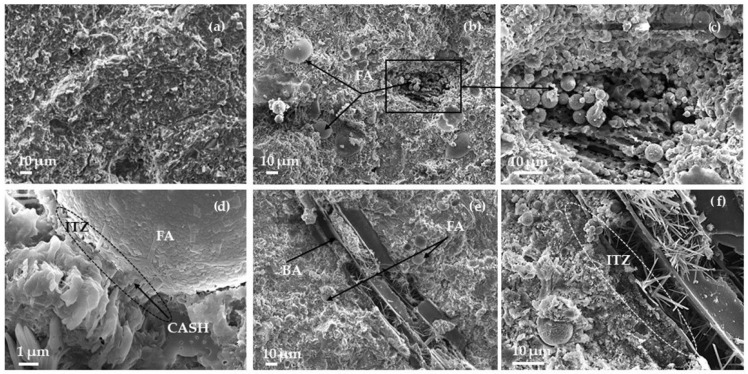
SEM images of FA0 (**a**), FA15 (**b**–**d**) and FAB15 (**e**,**f**).

Evaluation of portlandite (Figure 5) at 28 days showed that the samples containing FA had similar amounts of portlandite amounts calculated by the same cement content. Pure coal combustion due to a relatively low amount of ash was not analysed and all mass loss in the 420–530 °C temperature range was attributed to portlandite decomposition. The addition of 5% FA increased the portlandite amount slightly. This increase suggests that FA may moderately intensify the hydration of cement minerals and the formation of portlandite due to the large surface area and water demand of FA. In Figure 5b it is seen that clusters of portlandite have formed near the FA surface. Other authors [59] established, that in the initial 7 days of curing, the hydration reactions of Portland cement dominate, leading to a continuous increase in calcium hydroxide content. This increase is due to the rapid hydration of tricalcium silicate (C_3_S) and dicalcium silicate (C_2_S), which generate both calcium silicate hydrate (CSH) and CH as primary products. After this period, the pozzolanic reaction of fly ash becomes more prominent. Fly ash, containing reactive silica and alumina, reacts with the available CH to form additional CSH. This secondary reaction gradually consumes the CH produced earlier, resulting in a decrease in its concentration. Delayed cement hydration due to the carbon content of BA, which may contain unburned coal (Figure 6e,f), could be one of the causes [60,61].

The amount of calcium carbonates increased 20% in the samples modified with BA in comparison with the control sample due to the high amount of carbonates in BA. It should be noted that high amounts of calcium carbonates were identified in the samples of all compositions because limestone-rich cement CEM II containing approximately 15% CaCO_3_ was used for the preparation of test specimens. Interestingly, the mass loss in the temperature range from 110 °C to 350 °C was quite similar in all compositions except for FAB15, which had a mass loss of 10% less. Therefore, the amounts of CSH and CASH formed in the samples containing only FA and in the samples containing FA and 5% BA were similar.

**Figure 7:**
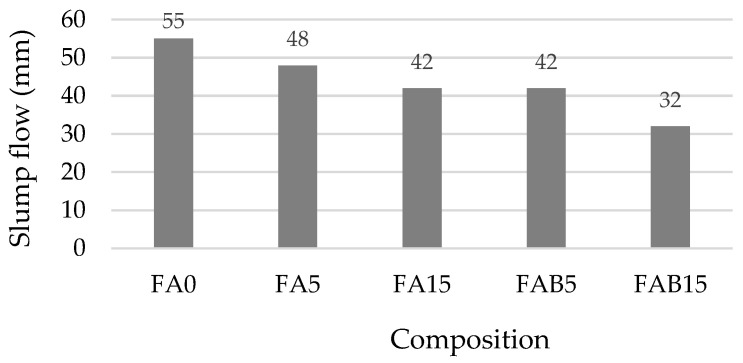
Slump flow of cement paste.

The SEM analysis (Figure 6) shows that after 28 days the densest structure is that of the reference sample (Figure 6a). FAs are distributed homogeneously (Figure 6b) and some of the FA agglomerates still exist (Figure 6b,c) due to which the increase in water absorption and the decrease in slump flow (Figure 7) was established. The interaction zone between the FA particles and the BA particles and cement matrix is dense, and various crystal hydrates (CH, CSH, and CASH) are growing on the surface of the particles. In the BA cavities of the coal particles there is intensive formation of ettringite (Figure 6e,f).

**Figure 8:**
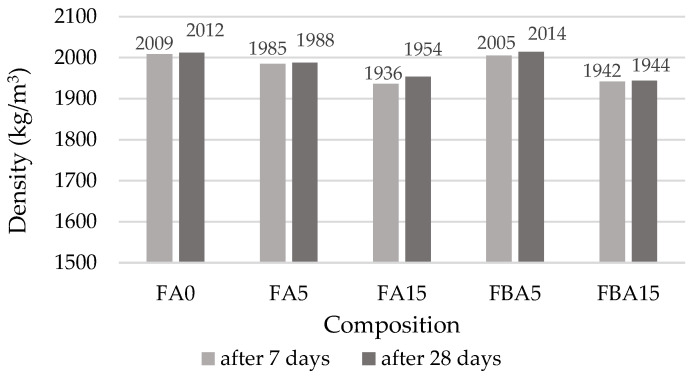
Density of hardened cement paste.

The density of hardened cement paste (Figure 8) decreased approximately 1% in the FA5 and FBA5, i.e., within the acceptable margin of error (the average standard deviation is 2–15 kg/m^3^); however, in the FA15 and FBA15 the density drop was approximately 4%. Higher water demand by BA caused a slight decrease in the density of the samples. The slump flow of FBA15 cement pastes (Figure 8) reduced approximately 40% and caused the development of a larger number of air voids. Other authors [12,62,63,64] found that a high level of cement replacement with BA significantly decreases the workability of concrete due to the porous structure of the ash.

**Figure 9:**
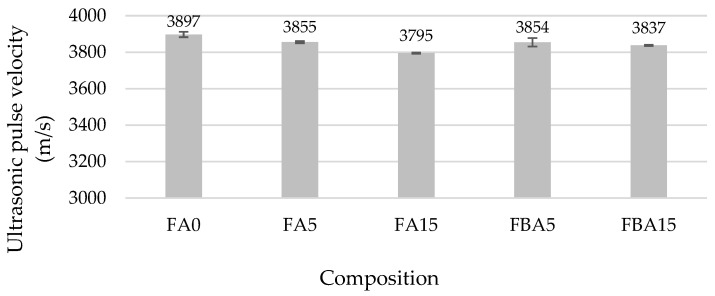
UPV of hardened cement paste.

The ultrasonic pulse velocity values also showed that the FA and BA added in respective amounts did not change the structure of the hardened cement paste samples (Figure 9). UPV in FA5 and FBA5 decreased only approximately 1% compared to the control sample. The greatest UPV decrease of 2.7% was observed in the samples where 15% of the cement was replaced with FA. A slight change in the structure of the specimens, in which up to 30% of cement was replaced with ash, could have been caused by the fixation of aluminate and silicate anions on the surface of the FA and BA, which resulted in the fixation of these materials with cement particles [65].

**Figure 10:**
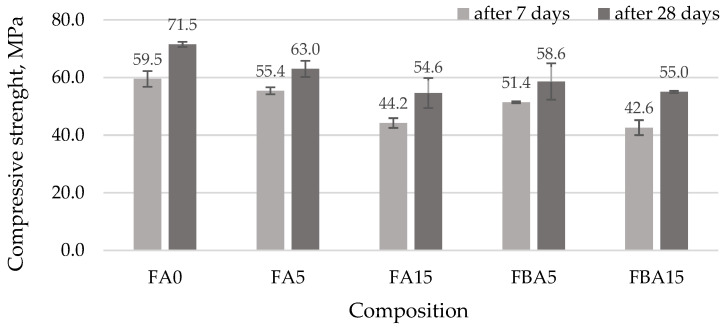
Compressive strength of hardened cement paste.

It is known that in the correlation between the UPV and compressive strength, when the UPV decreases, indicating lower density and potential defects, the compressive strength of the cementitious materials also tends to decrease. This is because the presence of gaps, cracks, and other imperfections within the cement matrix directly affects its ability to bear loads [66]. The compressive strength of hardened cement paste samples (Figure 10) decreased significantly. When 5% of the cement was replaced with FA, the compressive strength decreased 7.4% at 7 days and 13.5% at 28 days. When cement was replaced with 15% FA, the compressive strength decreased 34.6% at 7 days and 31% at 28 days. A similar drop in strength was also observed in FBA5 and FBA15 samples: 15.8%; 22.0%, 39.7% and 30%, respectively. This reduction in strength is primarily caused by the inferior mechanical properties of both FA and BA compared to cement. Furthermore, the increased porosity of these ashes leads to higher water absorption from the surrounding cement paste, resulting in a weaker interfacial transition zone and higher porosity. In the initial curing stages, the pozzolanic reaction is minimal due to the limited availability of Ca(OH)_2_, which hinders the development of additional strength. Additionally, the presence of FA and BA reduces the alkalinity of the pore solution, which in turn delays the hydration process [26]. Although FA and BA possess pozzolanic properties, their larger particle size results in delayed pozzolanic activity. This is evidenced by the initial reduction in strength observed at 7 and 28 days, but could increase at 90 days [12]. Some authors [27,28] found that the higher the BA replacement ratio, the higher the porosity and the lower the compressive strength at 28 days.

### 3.2. Exothermic Temperature Testing Results

**Figure 11:**
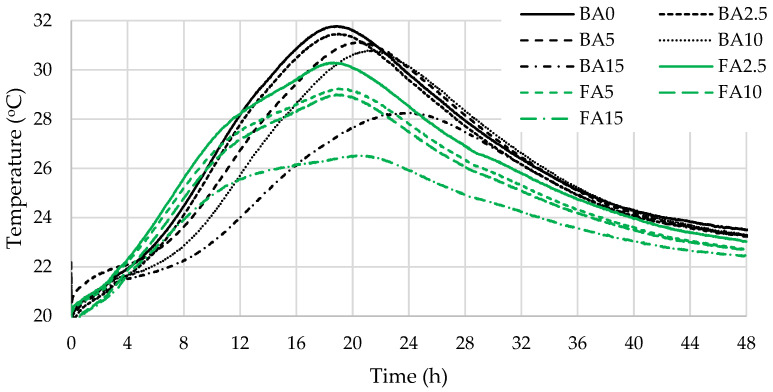
Exothermic temperature testing results.

Temperature monitoring tests (Figure 11) showed that in the reduced cement content both types of ash lower the exothermic temperature by 0.1–5 °C but have a different effect on the cement hydration time. BA added at 2.5% does not have an effect on the cement hydration rate, but the increase in BA to 5% and more decelerates cement hydration by approx. 2 h. 15% of BA delays cement hydration even more and the exothermic peak is reached about 5 h later. Changes in the exothermic curve of hydration were observed in samples modified with FA. Two peaks were identified, the first related to C_3_S hydration and the second, even higher peak, indicating the C_3_A hydration due to active aluminium in FA [1,67]. More aluminate phases formed in FA-modified specimens and caused the heat release at a later stage. The first peak appeared after about 9 h and the second peak appeared after 19–21.5 h, depending on the amount of cement replaced with FA. The authors Berra et al. [68] also determined that the higher the replacement level of cement with FA, the longer the initial and final setting times. The article [69] describes the direct relationship between the reduced heat release, or temperature in our case, and the lower early compressive strength of hardened cement paste.

### 3.3. Physical Mechanical Properties and Durability of Concrete

**Figure 12:**
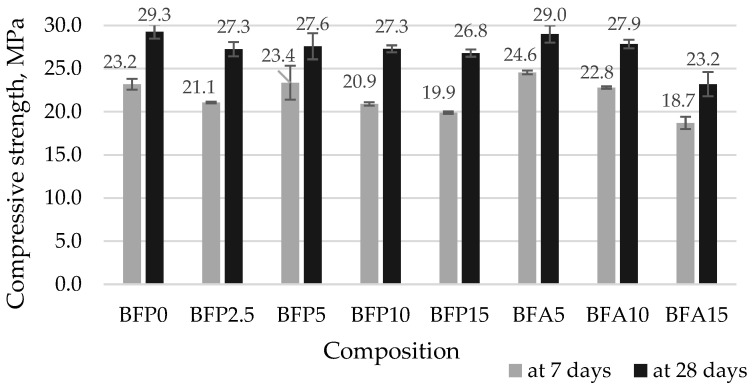
Compressive strength of concrete specimens at 7 and 28 days.

Figure 12 illustrates the influence of FA and BA on the compressive strength of concrete at 7 and 28 days. At 7 and 28 days the control specimens had the compressive strength of 23.2 and 29.3 MPa, respectively. The compressive strength values in the specimens containing 2.5% ash reduced by 9.1% and 6.8% compared to the reference. The tests showed that at 7 days the compressive strength of the samples containing 15% ash reduced by 14.2% and at 28 days the strength reduced by 8.5%. The samples with 5% FA showed the best strength results at 28 days. The strength of these samples was 29.0 MPa, that is, the same as that of the control sample. The analysis of strength changes at 7 and 28 days indicates the trend of increased compressive strength with a longer curing time due to pozzolanic reactions, and, therefore, there is a probability of a further increase in compressive strength with time [10]. According to Iyer and Scott [70], a higher level of reactivity in fly ash is associated with a more disordered structure, which is the result of variables such as changes in temperature, duration of combustion, and quenching during the fly ash manufacturing process.

**Figure 13:**
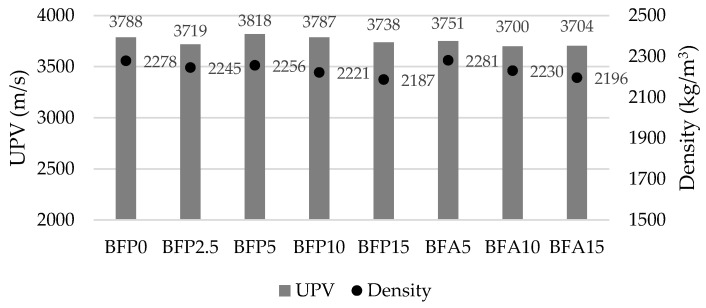
UPV and density of concrete.

Ultrasonic pulse velocity is one of the non-destructive methods for assessing concrete structure. The results presented in Figure 13 show that the density of concrete prepared with different amounts of ash reduces by up to 4% (changes from 2187 to 2281 kg/m^3^), whereas UPV changes insignificantly from 3700 to 3818 m/s compared to the control sample (3788 m/s). Other researchers [71] also found that the compressive strength and density of the samples with a higher BA content reduced due to the lower cement content in the samples. Malkit Singh [72] found that more uniform and homogeneous concrete can be obtained using coal bottom ash to replace natural fine aggregate.

**Figure 14:**
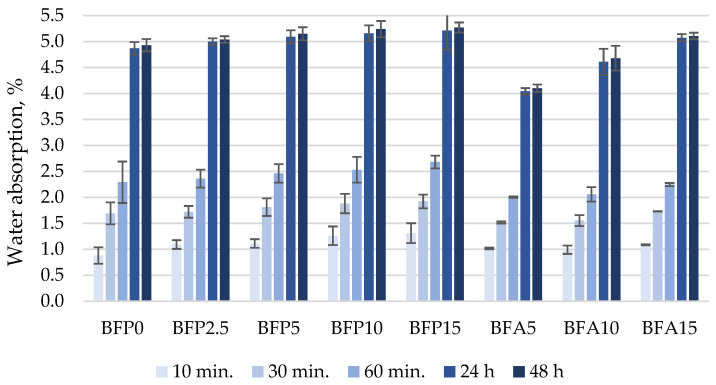
Water absorption kinetics of concrete specimens.

The highest water absorption rate (Figure 14) was observed in the BFP15 composition with the highest ash content (15% FA and 15% BA). During the first 10 min, the water absorption rate was 1.3%; after 30 min it was 1.9%, after 60 min it was 2.7%, after 24 h it was 5.2%, and after 48 h it reached 5.3% by mass of the specimen. The analysis of water sorption kinetics showed that the water absorption rate gradually increased with increasing FA content in the samples. The water absorption rate decreased only in samples BFA5 and BFA10 where only cement was replaced with 5% and 10% of FA. The decrease was caused by the pozzolanic reaction and the new formation of CSH, which filled part of the open pores and capillaries. Obvious changes were observed in the BFA5 samples: after 24 and 48 h the water absorption rate was reduced by 18.4% and 16.3% compared to the reference. Singh and Siddique [26] obtained similar results showing that coal bottom ash increased water absorption at an early age of concrete, but after a longer curing time, the water absorption rate became similar to that of the reference without coal bottom ash.

Table 5 illustrates the change in open and closed porosity according to the ash content in a concrete composition. The data in the table show that with the increase in FA and BA up to 15% the open porosity changes significantly from 11.23% to 11.64%, the total porosity changes within a broader range from 15.62% to 19.01%, and the closed porosity ranges from 4.38% to 7.49%. The freeze–thaw resistance factor Kf ranged from 4.34 to 7.22. The highest value of the freeze–thaw resistance factor and the predicted resistance to freezing and thawing cycles are specific to the samples BFA5, BFA10 and BFA15 modified only with FA added by weight of cement. Open pores and capillaries are formed when free water is removed from concrete. The voids have a negative effect on the resistance of concrete to freezing and thawing cycles, and the number of pores and their size especially depend on the water/cement ratio and the additives used. Closed pores are formed due to air entrainment, the contraction of hardening cement paste and CSH formation [73].

The results of the freeze–thaw resistance test are presented in Table 6. The compressive strength after 150 freeze–thaw cycles was reduced in the samples of all compositions. Only the BFP2.5 samples withstood 150 cycles according to the requirements of the standard, allowing for a 5% reduction in compressive strength, and only these specimens complied with the freeze–thaw resistance class F150. Although no cracks were identified in concrete specimens after the test, the compressive strength reduced from 4.7% to 21.8%. Specimens BFP2.5, BFP5, BFP15 and BFA10 demonstrated similar or better results than the reference. Therefore, it can be concluded that both FA and BA added at appropriate levels can improve the freeze–thaw resistance of concrete, especially when cement and aggregate are replaced at low levels, up to 2.5% by weight, and both types of ash are used in combination. Sample BFA10, where only 10% of cement was replaced with FA, showed one of the least reductions in compressive strength. The resistance to freeze–thaw is most often improved when pozzolanic additives that promote CSH formation are used.

**Figure 15:**
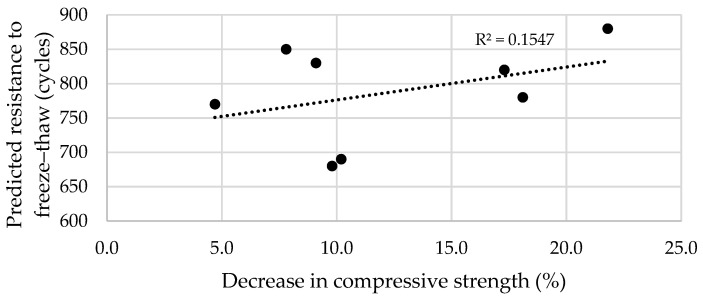
Correlation of predicted resistance to freeze–thaw cycles and respective decrease in compressive strength.

It should be noted (Figure 15) that there is no relationship between the predicted resistance to freeze–thaw cycles and the reduction identified in compressive strength because the coefficient of determination R^2^ is approximately 0.15, whereas a weak relationship according to the literature [74] is indicated by the coefficient value of 0.25. One of the reasons for the low coefficient value is the accelerated method with a NaCl solution, which was used to evaluate the resistance to this salt. Therefore, it can be concluded that FA and BA added at appropriate amounts can improve the resistance of concrete to freeze–thaw cycles and NaCl.

## 4. Conclusions

This study confirms the positive synergistic effect of fly ash and bottom ash in concrete mixtures, where FA replaces 15% of the cement and BA replaces 15% of the sand. This substitution strategy results in an approximately 15% reduction in CO_2_ emissions associated with cement production and a 15% reduction in the demand for natural sand aggregate. Modified concrete maintains properties comparable to control specimens, with a compressive strength from 29.3 MPa to 26.8 MPa and a decrease in strength loss after freeze–thaw cycles lower by 0.7% compared to the reference sample. On the contrary, the use of only FA without BA addition leads to a more significant compressive strength reduction up to 23.2 MPa and a substantial 17.3% reduction in compressive strength after 150 freeze–thaw cycles in a salt solution.

XRD tests showed that FA and BA do not change the qualitative conventional mineral composition of cement-based materials. However, the samples with a higher FA and BA content (15%) demonstrated intensive quartz peaks due to the high SiO_2_ content in the ash, 54.6% and 20.2%, respectively.

In terms of portlandite content, TG and DTG analyses showed that FA-modified samples had a similar amount of portlandite calculated by the same weight of hydrated cement. However, with the addition of FA at 5%, the amount of portlandite increased slightly. With the addition of BA to FA, the amount of portlandite calculated by the same weight of hydrated cement was reduced due to the slower cement hydration caused by the unburned coal present in BA and a higher amount of calcium carbonate.

BA added at 2.5% does not affect the cement hydration rate, but when the BA content increases to 5% or more, it delays hydration by approximately 2 h. A significant delay in hydration (5 h) was observed when 15% cement was replaced with BA; therefore, BA was used to replace part of the fine aggregate sand in concrete compositions.

FA and BA did not have a significant effect on the density and ultrasonic pulse velocity in hardened cement paste, but the added ash had a significant effect on the compressive strength of hardened cement paste, which reduced from 7% to 40%, especially at 7 days of curing. After a longer curing time, the decrease in compressive strength slowed due to pozzolanic reactions. FA and BA did not have such a significant effect on the compressive strength of concrete where coarse aggregates and fibres were used. The compressive strength reduced by only 10% in the sample where 15% cement was replaced with FA and 15% sand was replaced with BA.

The freeze–thaw test with NaCl solution showed that concrete samples modified with both FA and BA added at 2.5% equally had the highest freeze–thaw resistance. Most of the specimens showed better predicted and tested freeze–thaw resistance results than the reference. It showed the positive synergistic effect of FA and BA on the freeze–thaw resistance of concrete.

The obtained results lead to the conclusion that part of the cement in concrete can be replaced with FA and part of the fine aggregate can be replaced with BA. The hydration of this modified concrete is slightly delayed, and the compressive strength is reduced by up to 10%. However, the density and ultrasonic pulse velocity remain similar, while the resistance to freeze–thaw cycles increases. In further work, it would be important to find additives that neutralise the negative effects of FA and BA, which could help improve the strength and durability of concrete by reducing the amount of cement and natural aggregates. It could be through various chemical or by-product mineral additives.

## Figures and Tables

**Table 1:** Chemical composition of CEM II, FA and BA (wt.%).

Compound	Composition		
	CEM II	FA	BA
SiO_2_	18.4	54.60	20.2
Al_2_O_3_	3.98	25.30	2.31
Fe_2_O_3_	3.24	4.97	1.46
CaO	66.2	2.14	10.9
MgO	3.46	1.80	1.71
SO_3_	2.64	0.37	0.78
K_2_O	1.16	2.80	3.41
Na_2_O	0.19	0.84	0.28
Cl	-	-	0.13
CO_2_	-	n.a.	56.1
TiO_2_	0.30	1.07	0.11
P_2_O_5_	0.07	0.55	2.13
BaO	-	0.15	0.05
SrO	0.09	0.07	0.02
Mn_3_O_4_	-	0.06	-
MnO	0.06	-	0.23
LOI	-	4.37	4.05

**Table 2:** Compositions of the cement pastes (wt.%).

Mix Designation	Cement	FA	BA	W/S
FA0	100	0	0	0.35
FA5	95	5	0	0.35
FA15	85	15	0	0.35
FBA5	90	5	5	0.35
FBA15	70	15	15	0.35

**Table 3:** Compositions for the exothermic temperature testing (wt.%).

Mix Designation	Cement	FA According to Cement	BA According to Cement	Sand	W/(C + FA + BA)
FA0	23.50	0	0	76.5	0.35
FA2.5	22.91	2.5	0	76.5	0.35
FA5	22.33	5	0	76.5	0.35
FA10	21.15	10	0	76.5	0.35
FA15	19.98	15	0	76.5	0.35
BA2.5	22.91	0	2.5	76.5	0.35
BA5	22.33	0	5	76.5	0.35
BA10	21.15	0	10	76.5	0.35
BA15	19.98	0	15	76.5	0.35

**Table 4:** Compositions of concrete mixes (kg/m^3^).

Mix Designation	Binder (Cement + FA)		BA	Sand	Crushed Granite	PP Fiber	SP	W/B
	Cement	FA						
BFP0	300	0	0	980	1000	4.0	3.0	0.55
BFP2.5	292.5	7.5	24.5	955.5	1000	4.0	3.0	0.55
BFP5	285	15	49	931	1000	4.0	3.0	0.55
BFP10	270	30	98	882	1000	4.0	3.0	0.55
BFP15	255	45	147	833	1000	4.0	3.0	0.55
BFA5	285	15	0	980	1000	4.0	3.0	0.55
BFA10	270	30	0	980	1000	4.0	3.0	0.55
BFA15	255	45	0	980	1000	4.0	3.0	0.55

**Table 5:** Porosity parameters and predicted resistance to freezing and thawing cycles of concrete specimens.

Designation of Concrete	Open Porosity Pa, %	Total Porosity Pt, %	Closed Porosity Pu, %	K_f_	Predicted Freeze–Thaw Resistance, Cycles
BFP0	11.23	15.62	4.38	4.34	680
BFP2.5	11.32	16.84	5.53	5.43	770
BFP5	11.62	16.43	4.81	4.60	690
BFP10	11.64	17.73	6.09	5.81	780
BFP15	11.52	19.01	7.49	7.22	830
BFA5	9.31	15.89	6.57	7.84	880
BFA10	10.41	17.59	7.17	7.65	850
BFA15	11.22	18.67	7.46	7.39	820

**Table 6:** Freeze–thaw resistance results.

Designation of Concrete	BFP0	BFP2.5	BFP5	BFP10	BFP15	BFA5	BFA10	BFA15
Change in compressive strength, %	−9.8	−4.7	−10.2	−18.1	−9.1	−21.8	−7.8	−17.3
Appearance of specimens	No visible cracks							
Number of cycles	150							

## Data Availability

All results are presented in the article.
